# Active involvement of people with lived experience of suicide in suicide research: a Delphi consensus study

**DOI:** 10.1186/s12888-023-04973-9

**Published:** 2023-07-11

**Authors:** Karolina Krysinska, Ingrid Ozols, Anna Ross, Karl Andriessen, Michelle Banfield, Martina McGrath, Bronwen Edwards, Jacinta Hawgood, Kairi Kõlves, Victoria Ross, Jane Pirkis

**Affiliations:** 1 Centre for Mental Health, Melbourne School of Population and Global Health, The University of Melbourne, Melbourne, VIC, Australia; 2 mentalhealth@work, Melbourne, VIC, Australia; 3 Centre for Mental Health Research, Australian National University, Canberra, ACT, Australia; 4 Roses in the Ocean, Brisbane, QLD, Australia; 5 The Australian Institute for Suicide Research and Prevention, School of Applied Psychology, Griffith University, Brisbane, QLD, Australia

**Keywords:** Co-production, Consumers, Delphi study, Guidelines, Lived experience of suicide, Suicide research

## Abstract

**Background:**

The importance and value of involvement of people with lived experience of suicide has been recognized in suicide research and prevention. Nonetheless, clear guidance on research collaboration and co-production is lacking. This study aimed to address this gap by developing a set of guidelines on active involvement of people with lived experience of suicide in suicide studies., i.e., conducting research with or by people with lived experience, rather than to, about or for them.

**Methods:**

The Delphi method was used to determine statements on best practice for the active involvement of people with lived experience of suicide in suicide research. Statements were compiled through a systematic search of the scientific and grey literature, and reviewing qualitative data from a recent related study conducted by the authors. Two expert panels: people with lived experience of suicide (n = 44) and suicide researchers (n = 29) rated statements over three rounds of an online survey. Statements endorsed by at least 80% of panellists of each panel were included in the guidelines.

**Results:**

Panellists endorsed 96 out of 126 statements in 17 sections covering the full research cycle from deciding on the research question and securing funding, to conducting research and disseminating and implementing outcomes. Overall, there was a substantial level of agreement between the two panels regarding support from research institutions, collaboration and co-production, communication and shared decision making, conducting research, self-care, acknowledgment, and dissemination and implementation. However, panels also disagreed on specific statements regarding representativeness and diversity, managing expectations, time and budgeting, training, and self-disclosure.

**Conclusions:**

This study identified consensus recommendations on active involvement of people with lived experience of suicide in suicide research, including co-production. Support from research institutions and funders, and training on co-production for researchers and people with lived experience, are needed for successful implementation and uptake of the guidelines.

**Supplementary Information:**

The online version contains supplementary material available at 10.1186/s12888-023-04973-9.

## Background

The active involvement of people who have a personal experience of using health, mental health and/or social care services and/or caring for someone who uses such services (i.e., “consumers”, “experts-by-experience” or “people with lived experience”) in service and policy development has been relatively well established [1–3]. More recently, there has been growing emphasis on conducting research *with* or *by* consumers, instead of *to*, *about* or *for* consumers [4]. This represents a paradigmatic shift from an assumption that professional experts, such as researchers and other specialists, are best placed to decide on the subject and the type of research that should be undertaken [5]. Despite the increasing popularity of the concept, there is lack of consensus regarding the terminology associated with consumer involvement and participation in research, and different terms have been used internationally, including “patient and public involvement”, “service user involvement”, “patient participation”, “collaboration”, “partnership”, and “co-production” [6, 7].

Informed by the original “ladder of citizen participation” concept [8], different models [9], frameworks [10, 11], and tools [12–14], describe the type of active involvement of consumers in health research. One of these is co-production encompassing co-planning, co-design, co-delivery, and co-evaluation [7]. In co-production, “consumers [are] involved in, or leading, defining the problem, designing and delivering the solution, and evaluating the outcome, either with professionals or independently” [7; p, 2).

Consumer involvement can have a positive impact on both the research process [15] and consumers, researchers and communities [16]. The positive impacts include enhanced quality and appropriateness of research questions and recruitment strategies, more consumer-focused interpretation of research data and better dissemination and implementation [15, 17, 18]. Consumers can gain confidence and additional life skills, feel more empowered and valued, while researchers can achieve new insights and a greater understanding of their research area and value the views of those who ‘live’ the topic being investigated [16, 17, 19].

On the community level, consumer involvement contributes to a deeper understanding of mental health conditions for researchers and improved knowledge of research for consumers [16]. Despite these advantages, consumer involvement in research is not free from challenges and pitfalls, such as tokenistic involvement, power struggles, and compromised scientific quality [18, 19]. These issues can be minimized by provision of appropriate training to consumers and researchers, careful planning, and by ensuring adequate funding [15].

The importance of active involvement of people with lived experience of suicide, which is a phrase used interchangeably to refer to “consumers” or ”experts by experience”, has been increasingly acknowledged in the field of suicide research and prevention. A lived experience of suicide is defined as “having experienced suicidal thoughts, survived a suicide attempt, cared for someone through suicidal crisis, or been bereaved by suicide” [20]. According to O’Connor and Portzky [21], “recognition of the importance of postvention and those with lived experience (…) is a positive development in the field in recent decades” and a “key to suicide research and prevention activities” (p. 4). Studies on the process and outcomes of consumer involvement in suicide research have been emerging [22–29].

To-date the literature has been focused on involvement of consumers or people with lived experience in research [13, 15, 16]. As suicide is understood as a multifactorial event and behavior [30], it is not clear whether health-related research guidelines and frameworks of “consumer involvement”, including co-production, can effectively guide active involvement of people with lived experience of suicide in suicide research. The Voice of people with Lived Experience of suicide (VocLE) study aimed to address this lack of clear guidance by developing a set of guidelines on active involvement (i.e., research *with* or *by* people with lived experience, rather than *to*, *about* or *for* them) in suicide research. The VocLE study was a 2-year mixed methods study conducted in Australia including a qualitative interview study and a quantitative expert consensus study. The guidelines have been developed using the Delphi expert consensus method, which is used to gather practice-based evidence where it is not feasible to determine best-practice through experimental research [31]. This method has been successfully used to develop guidelines on consumer involvement in medical and social research [13, 32, 33]. Our paper reports on the process of developing guidelines on active involvement of people with lived experience of suicide in suicide research and presents the resulting set of statements.

## Methods

### The Delphi method

We used the Delphi consensus method, as applied to mental research [31] to determine statements on best practice for the active involvement of people with lived experience of suicide in suicide research. The Delphi consensus method is effective for establishing an evidence-base in the absence of literature on the topic. The Delphi method involves three stages: (a) sourcing statements, (b) development of the Delphi survey, and (c) the formation of an expert panel who complete the survey over three rounds [31].

#### 1. Sourcing statements

We conducted a systematic search of the scientific and grey literature to source statements about active involvement of people with lived experience of suicide in suicide research. Researchers KKr and KA searched Medline and PsycINFO databases. Medline was searched with a combination of MeSH (Medical Subject Headings) and text words: (suicide research.mp (multi-purpose). OR Suicide/ OR Suicide.mp. OR mental health research.mp. OR psychiatric research.mp. OR disability research.mp.) AND (consumer participation.mp. OR user participation.mp. OR carer participation.mp. OR patient participation.mp. OR Patient Participation/ OR consumer involvement.mp. OR user involvement.mp. OR carer involvement.mp. OR patient involvement.mp. OR lived experience.mp.) AND (guide*.mp. OR Practice Guideline/ OR Guideline/). The same search string was used in PsycINFO. The search was limited to English language publications, and research published within the past 10 years (from 2012 to the time of the search in May 2021).

Using similar combinations of the search terms used to search the scientific databases, researcher KKr searched the grey literature using Google search engines of English-speaking countries (Google.com, Google.com.au, Google.co.uk, Google.nz, Google.ca). Google was searched by combining search words (i.e., a total of 24 combinations) from each of the three groups: (a) “suicide research”, “mental health research”, “disability research”, (b) “consumer participation”, “user participation”, “carer participation”, “consumer coproduction”, “user coproduction”, “carer coproduction”, “service users”, “lived experience”, and (c) “guide*”. We used Google Chrome in incognito mode to avoid potential bias from the search history. Researcher KKr screened the first 20 results of each search for relevant content. Of the total results screened, 14 resources were determined to be relevant (Appendix 1).

In addition, researchers AR, IO, KKr, KKo, MMG, and VR read the deidentified transcripts and extracted the relevant statements on active involvement of people with lived experience of suicide in suicide research from a total of 36 individual interviews with people with lived experience of suicide (n = 19) and suicide researchers (n = 17). The qualitative interviews were conducted in the context of the mixed-methods VocLE study to obtain the views of people with lived experience of suicide and suicide researchers on involvement of lived experience in suicide research. The findings will be published elsewhere.

#### 2. Development of the Delphi survey

Statements, based on the literature and the interviews, were compiled into a Delphi survey. Four researchers (AR, KA, KKr), including a lived experience researcher (IO), examined the statements to ensure each contained a separate idea and was within the scope of the study. Where necessary, statements were rephrased to improve clarity, ensuring each statement about how people with lived experience of suicide can be involved in suicide research was clearly described.

Following this, other team members (JH, KKo, VR), including three lived experience researchers (BE, MB, MMG), reviewed and approved the statements for inclusion in the survey. The statements (N = 114) were grouped into 17 sections: (1) Research Institutions; (2) Collaboration and Co-production; (3) Developing Collaborative Networks; (4) Representativeness and Diversity; (5) Managing Expectations; (6) Time, Budgeting and Other Resources; (7) Training; (8) Language; (9) Communication and Shared Decision Making; (10) Sharing Power; 11) Deciding on the Research Question; 12) Conducting Research; 13) Support and Self-care; 14) Self-disclosure, Multiple Roles and Conflict of Interest; 15) Acknowledgement; 16) Monitoring and Evaluation; and 17) Dissemination and Implementation.

As the VocLE study was funded to be conducted in Australia, the key terms “lived experience of suicide”, “lived experience researcher”, “academic researcher”, and “co-production” were defined in the introduction to the online survey based on current practice in suicide prevention and research in Australia and the literature (Table 1).

**Table 1:** Definitions of key terms used in the Delphi expert consensus study

Term	Definition	Reference
Lived experience of suicide	Having experienced suicidal thoughts, survived a suicide attempt, cared for someone through suicidal crisis, or been bereaved by suicide.	[20]
Lived experience researcher” (also “consumer academic” or “lived experience in research”)	A paid academic role, whether as an employee, contractor consultant, or advisor. While lived experience researchers are involved as advisors in other’s research, as partners in collaborative research, or as leaders in research, they draw on their lived experience of suicide to promote and enable the engagement of people with lived experience of suicide in all stages of research	[35, 37–39]
Academic researcher	A scholar, teacher, researcher in a university or other institute of higher education. An academic researcher may have disclosed or not disclosed relevant lived experience.	[35, 36]
Co-production	People with lived experience of suicide are either involved in or defining the research question (co-planning), designing (co-design) and delivering the solution (co-implementation), and evaluating the outcome (co-evaluation), either with professionals or independently.	[7]

#### 3. Formation of the Delphi expert panel

Two Delphi expert panels were formed: (a) people with lived experience of suicide who had experience of, or a particular interest in, conducting or participating in suicide research and (b) people who undertook suicide prevention research as part of their professional role (suicide researchers). People with lived experience of suicide could participate if they: (a) were aged 18 and over, (b) had lived experience of suicide (i.e., survived a suicide attempt more than 6 months prior to participating in this study, were bereaved by suicide more than 6 months prior to participating in this study, had experienced suicidal thoughts, and/or had been caring for someone through a suicidal crisis), (c) have ever participated as a co-researcher, an advisor, a participant in a research project in regards to suicide/prevention, and/or (d) had an interest in participating in suicide research studies in the future. Suicide researchers could participate if they have been actively involved in suicide-related research in Australia [34].

We recruited people with lived experience of suicide through various sources. ACACIA: The ACT Consumer & Carer Mental Health Research Unit at the Lived Experience Research Unit (Australian National University), the Australian Institute for Suicide Research and Prevention (AISRAP, Griffith University), and Roses in the Ocean, disseminated the study announcement to their members. People with lived experience of suicide who had taken part in the qualitative interviews and had consented to be informed about the Delphi study also received the study announcement. In addition, we posted the study announcement on social media (Facebook, Twitter, LinkedIn).

Suicide researchers were recruited through the Suicide Prevention Researcher Network at the Centre for Mental Health at the University of Melbourne (“LIFEWAYS Project: Leading research into suicide prevention”; previously known as the National Leadership in Suicide Prevention Research Project), a register held by the Australian National University Lived Experience Research Unit, the researcher network at AISRAP, and study announcements posted on social media (Facebook, Twitter, LinkedIn). Suicide researchers who had taken part in the qualitative interviews and had consented to be informed about the Delphi study also received the study announcement.

Data regarding professional research discipline or setting were not collected. All panellists provided online informed consent before participating in the study and were offered a $AUD15 gift voucher to reimburse them for their time for completing each round of the online survey.

#### 4. Delphi consensus survey rounds

Data collection occurred between November 24, 2021, and March 27, 2022. The Round One survey included sociodemographic questions and 114 statements to be rated by the expert panellists. The survey was completed via Qualtrics, a secure online platform for survey hosting. To complete the survey, expert panellists rated each statement as “essential”, “important”, “don’t know/depends”, “unimportant” or “should not be included” in the guidelines. In Round One, free text boxes were also included at the end of each section of the survey to allow panellists to provide comments about the statements or suggest additional statements. These comments were read and screened for any ideas that had not already been incorporated into the survey. Ideas that were determined to be original were developed into new statements (n = 12) and were introduced in the Round Two survey. Statements that met the criteria to be re-rated in the Round One survey (that is, were rated by 70–79.9% of both expert panels as “important” or “essential” or rated by 80% or more of one expert panel as “important” or “essential”) were also included in the Round Two survey. Statements that were newly added in Round Two based on panellist feedback and met the criteria to be re-rated comprised the Round Three survey.

### Data analysis

Data were analyzed in Microsoft Excel. Demographic characteristics of the two panel groups were summarized using descriptive statistics. The decision to have the two panels was based on the number of panellists recruited for the study being high enough to ensure stability in ratings (i.e., one panellist would not have too much influence on the outcome of an item in the separate panel groups). A benefit of having two stable panel groups is that any differences in responses can be contrasted and compared, allowing a better understanding of each expert group, rather than this being lost in the ratings if they are pooled from one big panel [31].

Endorsement ratings were calculated for each panel by adding the percentage of panellists rating the statement as “important” or “essential” which determined the outcome of each statement. Statements that were rated by 80% or more of panellists as “essential” or “important” across both panels were endorsed as actions to be taken to involve people with lived experience of suicide in suicide resarch. Statements that were rated by 70–79.9% of panellists in both panels, or by 80% or more of panellists in one panel only, as “essential” or “important” were re-rated in the following survey round. Statements that were rated by less than 70% of panellists in both panels, or 70.0–79.9% of panellists in one panel only, as “essential” or “important” were excluded. When re-rated in the following round(s), statements that again met the criteria to be re-rated (as described above) were considered to not have achieved consensus regarding inclusion or exclusion of the statement within two rounds and were excluded. Trends in ratings between the two panels were analysed more broadly across survey sections and in relation to more specific topics by comparing and contrasting endorsement rates.

### Ethics approval

This study received Human Research Ethics Committee approval from The University of Melbourne (HREC# 22453).

## Results

### Expert panels

The study included two panels: a lived experience panel and a researcher panel. The expert panels comprised a total of 73 panellists, including 44 people with a lived experience of suicide and 29 suicide researchers, 14 (48.3%) of whom also self-identified as having lived experience of suicide. Panellists’ characteristics are described in Table 2.

**Table 2:** Panellists’ characteristics (N = 73)

	N	n (% Female)	Age M years (SD), range
People with lived experience of suicide	44	32 (72.7%)	52.1 (13.9), 19–75
Suicide researchers	29	22 (75.9%)	42.9 (12.0), 24–68

The overall panellist retention rate was high, with over 80% of panellists who completed Round One also completing Round Two and Round Three. Panellist retention was stronger amongst researcher panellists as 100% of suicide researchers who completed the Round One survey also completed the Round Two and Round Three surveys (Table 3).

**Table 3:** Participation of Delphi panellists in each survey round

	Round One n	Round Two n (% of Round One)	Round Three n (% of Round One)
People with lived experience of suicide	44	33 (75.0%)	30 (68.2%)
Suicide researchers	29	29 (100.0%)	29 (100.0%)
Total	73	62 (84.9%)	59 (80.8%)

### Delphi consensus surveys

**Fig. 1:**
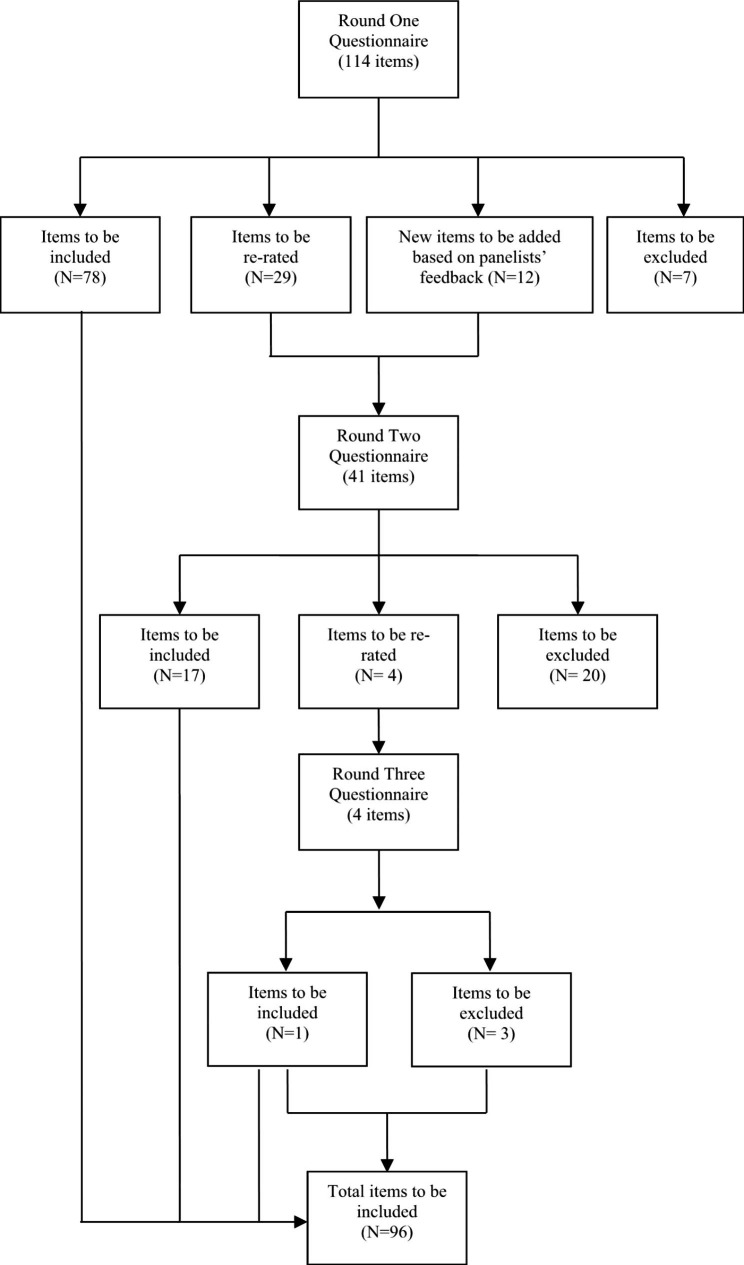
Overview of statements throughout the three survey rounds

Figure 1 presents a flow chart of statements ratings across the three Delphi rounds. A total of 126 statements were rated across the three survey rounds, resulting in 96 statements (76.2%) endorsed as important or essential for actively involving people with lived experience in suicide research, and 30 statements (23.8%) being rejected. Table 4 presents statements included in the guidelines. The endorsement ratings for each statement across the survey rounds are provided in Appendix 2.

**Table 4:** Included statements (n = 96) endorsed by both Delphi expert panels

Section	Statements
Research Institutions	Research institutions should: 1. Commit to involve LE researchers in the research process. 2. Establish LE advisory committee/s. 3. Embed co-production in the organisation’s culture, from the leadership team down. 4. Develop a policy on LE researcher involvement in research, defining possible roles LE researchers may play, and their level of involvement. 5. Ensure that ethics committees are informed of and familiar with co-production in suicide prevention research. 6. Build capacity of all researchers, including LE, through training, mentoring and support. 7. Plan budget strategies and allocate funds to support, implement and acknowledge LE researchers’ involvement. 8. Build in the time required to co-produce properly and to educate funders of research that this is an integral part of the research process. 9. Embed involvement of LE researchers in all stages of the research cycle and processes, i.e., deciding what and how to research, doing research, communicating research results, and what next steps might be expected in the research project. 10. Ensure that LE researcher(s) have a clear pathway to professional development. 11. Provide appropriate training/support opportunities so that LE researchers feel comfortable taking leadership positions within a research team.
Collaboration and Co-production	12. Both academic and LE researchers should support building wider knowledge of what LE researchers can bring to a research project among proposed participants of projects, e.g., offering a choice of an interview by LE researcher or a non-LE researcher working on the project. Academic researchers should: 13. Possess the knowledge and understanding of what co-production is and all stages of co-production approach: co-planning, co-design, co-implementation, and co-evaluation. 14. In collaboration with LE researchers recognise what is and what in not co-production. 15. Embed co-production from the outset of the research project. 16. Advocate for policy change towards embedding LE collaboration and co-production within universities and funding bodies. 17. Seek out training, support, and mentoring from lived experience organisations in relation to co-production in research.
Developing Collaborative Networks	Academic researchers should: 18. Invest time building relationships and developing networks with LE researchers and LE organisations, e.g., to allow for sharing of knowledge between LE organisations and research environments. 19. Acknowledge when approaching people with LE or LE organisations, that not all wish to become involved in research. 20. Showcase research projects where LE involvement has made a demonstrable impact. 21. Keep a record of LE people with relevant experience and expertise. 22. Clearly communicate why and how LE researchers can contribute to research, as this may not be immediately understood.
Representativeness and Diversity	Academic researchers should: 23. Recognize the lived experience (whether bereavement, suicide attempt, suicidal ideation, caring for a suicidal person), abilities and cultural backgrounds required for specific research projects will vary depending on the focus and topic. 24. Ensure selection and inclusion criteria of LE researchers are equitable, skill- and merit- based and related to project topic. 25. Use the full range of available networks to involve LE researchers from the so-called ‘hard to reach’ groups, who may not otherwise be involved, e.g., due to socio-economic, cultural or circumstantial disadvantage. 26. Ensure that they get a diversity of relevant LE views and perspectives. 27. Recognise LE researchers can wear ‘several hats’ due to their professional background, unique skills, and their contributions to research may be broad.
Managing Expectations	Academic researchers should: 28. Ensure roles and responsibilities of academic and LE researchers are initially clearly defined and agreed as far as possible, with the note that this may evolve over the course of the project. 29. Clearly communicate the realities of funding with LE researchers, such as potential for a project not being funded. 30. Negotiate the different or competing priorities, needs, agendas, and outcomes of academic and LE researchers. 31. Ensure that timelines, boundaries, and confidentiality requirements are known and understood by academic and LE researchers.
Time, Budgeting and Other Resources	Academic researchers should: 32. Be aware of the financial and time costs of conducting genuine LE research involvement and factor these into the budget and timeframe of the project in advance. 33. Ensure office and educational infrastructure/ practical supports are accessible to LE researchers. 34. Budget for the education and training of co-production methodologies by appropriate facilitators with LE coproduction experience. 35. Promptly reimburse LE researchers for any expenses incurred through their involvement in research in line with the funding or any contractual agreements, such as travel, training, conference attendance. 36. Ensure that the method and structure of payment is appropriate to the level and type of LE researcher’s involvement.
Training	37. Both LE and academic researchers should be trained in principles and philosophy of co-production. 38. Training for academic and LE researchers should be delivered collaboratively. Lived Experience researchers should: 39. Be provided with sufficient training and support opportunities for appropriate skill development to perform for their role. 40. Receive training and support on how to effectively engage with research, e.g., training on a topic area and process of research. 41. Receive information and support regarding questions about ethics, privacy, and confidentiality in research. Academic researchers should: 42. Learn from academic mentors and colleagues experienced in co-production. 43. Receive training in safe and appropriate language around suicide. 44. Receive training in mental health literacy, if it is relevant to the type of the research they do (e.g., qualitative data collection). 45. Receive training in trauma-informed practice, if it is relevant to the type of research they do (e.g., qualitative data collection).
Language	46. Academic and LE researchers should agree on terms/language relevant to the research project. Academic researchers should: 47. Ensure that language used is accessible, safe, and understandable to academic and LE researchers. 48. Use language/terms LE researcher is comfortable with, i.e., person with lived experience, consumer, survivor, client. 49. Present research data in an understandable way.
Communication and Shared Decision Making	Academic researchers should: 50. Ensure all forms of communication, e.g., in person and online, are appropriate and accessible for academic and LE researchers. 51. Ensure everyone involved in the research project has appropriate information and support to feel confident in decision making. 52. Work out collaboratively at the beginning of the project the process for and the frequency of regular check ins regarding how everyone is feeling about shared decision making. 53. Communicate clearly and regularly with LE researcher(s), particularly if not in ‘regular’ workplace attendance. 54. Provide regular email updates to all members of the research team, including LE researchers. 55. Be flexible in terms of meeting dates, times, location, and numbers. 56. Hold meetings in a location where LE researcher(s) feel(s) comfortable. 57. Both academic and LE researchers should avoid a ‘us against them’ dynamic in the research team.
Sharing Power	Academic researchers should: 58. Collaboratively address the power differential between the academic and LE researchers at the outset of a research project. 59. Acknowledge and value the knowledge and expertise a LE researcher can contribute. 60. Recognise that sharing power within co-production demands critical reflective practice, and consistent attention to fluctuating power relations. 61. Understand and trust the choice of a LE researcher to be involved in a research project. 62. Understand that exclusion of LE researcher(s) for fear of risk is devaluing. Lived Experience researchers should: 63. Acknowledge and value the knowledge and expertise an academic researcher can contribute, such as research design and research evidence-base. 64. Recognise that the voice of the academic researcher is equal to others in the design group or leading the study.
Deciding on the Research Question	Academic researchers should: 65. Conceptualise and brainstorm research needs and questions and identify research gaps together with LE researchers. 66. If the research questions have already been decided, researchers should be clear and open about other opportunities for input from LE researcher(s). 67. Encourage LE researchers to approach academic researchers with an idea for a research topic. 68. Understand that sometimes it is difficult for people with LE who are unfamiliar with research to identify research questions. It may help to first discuss ideas, problems to be solved that people with LE experience, before discussing how these might be turned into research questions.
Conducting Research	Academic researchers should: 69. Consider the type of LE researcher’s engagement appropriate in a particular project, a particular task within a project, and across different types of research activities. 70. Include LE researcher(s) in discussions about recruitment as they can provide valuable insight into appropriate ways to reach vulnerable and/or minority groups. 71. Invite LE researcher(s) to be involved in discussions about research findings as they may be able to provide an understanding of unexplained or unusual results. 72. Ensure that the LE engagement is appropriate by considering both the skills of the people offering the engagement opportunity (i.e., researchers) and skills of the LE being engaged. 73. Make it desirable for LE researchers to participate in research, e.g., by emphasising the value of LE researchers to provide insider knowledge, expertise, and perspective that could change the way research is conducted.
Support and Self-Care	Academic researchers should: 74. Recognise that exposure to explicit LE stories can be distressing for them and/or contribute to their vicarious traumatisation. 75. Have access to support and supervision to prevent/minimise their vicarious traumatisation. 76. Openly discuss with LE researchers any risks resulting from the co-production process, e.g., a clear plan should be in place that describes how to respond to the emotional distress of team members. 77. Acknowledge that LE researchers can make a choice (i.e., dignity of risk) and autonomy in making the determination to contribute their expertise to research. 78. Provide adequate support for LE researchers on three levels: (a) emotional support (e.g., access to de-briefing, mentoring or peer support), (b) practical support (e.g., assistance with travel arrangements or managing out of pocket expenses), and (c) research support (e.g., training and supervision in research practices). 79. Discuss openly the possibility of the LE researcher(s) becoming ill or unable to work and make clear arrangements in case of this happening. Lived Experience researchers should: 80. Prioritise their wellbeing and ensure appropriate self-care. 81. Have a self-care management plan in place in case they are triggered by talking about their LE.
Self-Disclosure, Multiple Roles and Conflict of Interest	Both Academic and Lived Experience researchers should: 82. Agree on when and how much to disclose their LE, and how to set appropriate boundaries. 83. Declare any existing conflicts of interest.
Acknowledgement	Academic researchers should: 84. Acknowledge the contribution LE researchers made to the research when writing journal articles and reports. 85. Enable joint authorship and contribution to research publications. 86. Agree with LE researcher(s) on Intellectual Property and who will own any information or products that has been generated together.
Monitoring and Evaluation	Academic researchers should: 87. Build in monitoring and evaluation mechanisms at the outset of the research study to learn from experience and inform future actions in terms of LE researcher(s)’ contribution. 88. Provide feedback on how a LE researcher has impacted on research, its progress, results, and outcomes. 89. Ensure that publications stemming from projects report on the methods used to engage LE researcher(s), such as who was involved and how, and on the outcomes of involvement. 90. Organise team meetings focused on reflective discussion about the research and co-production process. 91. Find a way to mark the ending of a project and a way to enable LE and academic researchers to reflect upon their experience and the learning they have gained through their collaboration.
Dissemination and Implementation	Academic researchers should: 92. In collaboration with LE researcher(s) plan dissemination strategy and opportunities for translating results and findings into policy and practice. 93. Provide LE researchers opportunities to lead on some of the dissemination and implementation activities, e.g., preparing plain language summaries of the research findings. 94. Collaborate with LE researcher(s) to develop plain language summaries of research results and findings. 95. Invite LE researcher(s) to co-present at academic conferences, presentations, and media briefings. 96. Seek LE researcher(s) input into report writing, the development of policy recommendations and/or translation plans.

LE: Lived Experience

In general, there was a substantial level of agreement between the two panels with a correlation of 0.63 in ratings in Round One. Table 5 summarizes the number of statements that were rated and endorsed in each survey section. Both panels in Round One of rating endorsed 78 statements (68.4%) and rejected seven statements (6.1%). Two statements were unanimously endorsed by the two panels: “lived experience researchers should receive information and support regarding research ethics, privacy and confidentiality” (section on Training) “academic researchers should present research data using understandable terms” (section on Language).

**Table 5:** Number of statements rated and endorsed by both panels across the three survey rounds

Section	N statements rated	N (%) endorsed	N (%) rejected
Research Institutions	14	11 (78.5%)	3 (21.5%)
Collaboration and Co-production	7	6 (85.7%)	1 (14.3%)
Developing Collaborative Networks	8	5 (62.5%)	3 (37.5%)
Representativeness and Diversity	9	5 (55.5%)	4 (44.5%)
Managing Expectations	6	4 (66.6%)	2 (33.4%)
Time, Budgeting and Other Resources	7	5 (71.4%)	2 (28.6%)
Training	13	9 (69.2%)	4 (30.8%)
Language	4	4 (100%)	0 (0%)
Communication and Shared Decision Making	9	8 (88.8%)	1 (11.2%)
Sharing Power	10	7 (70%)	3 (30%)
Deciding on the Research Question	5	4 (80%)	1 (20%)
Conducting Research	6	5 (83.3%)	1 (16.7%)
Support and Self-Care	11	8 (72.7%)	3 (27.3%)
Self-Disclosure, Multiple Roles, and Conflict of Interest	3	2 (66.6%)	1 (33.4%)
Acknowledgement	4	3 (75%)	1 (25%)
Monitoring and Evaluation	5	5 (100%)	0 (0%)
Dissemination and Implementation	5	5 (100%)	0 (0%)
TOTAL	126	96 (76.2%)	30 (23.8%)

Eight statements were rejected by both panels in the online survey (Table 6). These included two statements in the section on Representativeness and Diversity, and one statement each in sections on practical ways Research Institutions can support engagement of lived experience researchers, Collaboration and Co-production, Developing Collaborative Networks, Conduct of Research, Communication and Shared Decision, and Sharing of Power.

**Table 6:** Excluded statements (n = 8) rejected by both Delphi expert panels

Section	Statement	Lived Experience panel (% endorsement)	Research panel (% endorsement)
Research Institutions	Research institutions should ensure lived experience researchers are included for supervision of post graduate research students, if feasible.	79.5	55.2
Collaboration and Co-production	Both academic and lived experience researchers should acknowledge that there may be research designs (e.g., ecological studies) where complete co-production may not be possible.	69.7	72.4
Developing Collaborative Networks	Academic researchers should explore the local community and identify local lived experience groups.	77.3	62.1
Representativeness and Diversity	Academic researchers should involve different lived experience researcher(s) at different stages of the research process and co-production, depending on the needs.	75	34.5
	Academic researchers should engage lived experience researchers not linked to the established lived experience organisations to ensure greater diversity.	79.5	48.3
Communication and Shared Decision Making	Academic researchers should ensure all individuals are accessible to each other, e.g., sharing everyone’s email and contact details.	59.1	51.7
Sharing Power	Academic researchers should consider an independent facilitator, rather than a researcher, to support the process of collaboration and ensure the person is suitable to everyone involved in the research.	61.4	37.9
Conducting Research	Academic researchers should ensure people with lived experience pilot research measures and processes before they are implemented in the research.	79.5	62.1

Note: Statements that were rated by less than 70% of panelists in both panels, or 70–79.9% of panelists in one panel only, as “essential” or “important” were excluded

There were consistent discrepancies between the lived experience and researcher panels throughout the Delphi process, resulting in rejection of 15 statements (11.9%). Throughout the process, the lived experience panel consistently endorsed (> 80%) and the researcher panel consistently rejected (< 80%) 14 statements (Table 7). These included three statements in the section on Training, two statements in the section on practical ways Research Institutions can support engagement of lived experience researchers, and two statements in the section on Representativeness and Diversity. The other seven statements were in sections on Developing Collaborative Networks, Managing Expectations, Time, Budgeting and Other Resources, Sharing Power, Deciding on the Research Question, Self-Disclosure, Multiple Roles and Conflict of Interest, and Acknowledgement (one statement in each section). Throughout the process, the researcher panel consistently endorsed (> 80%) and the lived experience panel consistently rejected (< 80%) a statement in the section on Support and Self-Care, which stipulated that lived experience researchers should be ready or in recovery to engage in the research process. There were no consistent discrepancies between the two panels regarding statements in the sections on Language, Communication and Shared Decision Making, Conducting Research, Monitoring and Evaluation, and Dissemination and Implementation.

**Table 7:** Excluded statements (n = 15) with differences in ratings between the two Delphi expert panels

		Round One		Round Two	
Section	Statement	LE panel (% endorsement)	Researcher panel (% endorsement)	LE panel (% endorsement)	Researcher panel (% endorsement)
Research Institutions	Research institutions should have designated lived experience leadership positions within research organizations.	93.2	75.9	84.8	79.3
	Research institutions should provide lived experience researchers with opportunities to network with other lived experience researchers.	90.9	79.3	93.9	79.3
Developing Collaborative Networks	Academic researchers should support information provision regarding lived experience focused research, knowledge, and opportunities, e.g., via newsletters.	90.9	75.9	100.0	72.4
Representativeness and Diversity	Academic researchers should involve more than one lived experience researcher in a research project.	86.4	51.7	90.9	31.0
	Academic researchers should involve and consult local lived experience organizations to be sensitive to local issues and challenges regarding suicide and suicide prevention.	93.9	69.0	90.00	65.52
Managing Expectations	Academic researchers should align research priorities, method choices, and approaches of academic and lived experience researchers.	86.4	79.3	90.9	79.3
Time, Budgeting and Other Resources	Academic researchers should contact their organization or university to see if they have any funding for lived experience researcher(s’) involvement, prior to the grant application being accepted.	86.4	69	84.8	55.2
Training	Lived experience researchers should receive specific training in the language and terminology used in research.	88.6	72.4	90.9	79.3
	Academic researchers should receive training in research language (e.g., no jargon or acronyms) that is safe and understandable for lived experience researchers.	95.5	79.3	97.0	79.3
	Academic researchers should seek insight into the lived experience of mental health issues through exposure via attending community centres, psychiatric in-patient wards or attending lived experience presentations, if it is relevant to the type of research they do (e.g., qualitative data collection).	86.4	51.7	93.9	58.6
Sharing Power	Academic researchers should recognize the voice of the lived experience researcher as equal to others in the design group or leading the study.	93.2	79.3	87.9	75.9
Deciding on the Research Question	Academic researchers should consult with lived experience groups and organizations about their priorities for research.	93.2	79.3	97.0	79.3
Support and Self-care	Lived experience researchers should be ready or in recovery to engage in the research process.	68.2	89.7	78.8	86.2
Self-disclosure, Multiple Roles, and Conflict of Interest	Both Academic and lived experience researchers should be transparent regarding multiple roles, e.g., an academic researcher with lived experience or a lived experience researcher involved in advocacy work.	93.2	72.4	87.9	65.5
Acknowledgement	Academic researchers should ensure research findings are available to public contributors, e.g., publish in open access journals.	88.6	79.3	90.9	79.3

Note: Statements that were rated by less than 70% of panelists in both panels, or 70–79.9% of panelists in one panel only, as “essential” or “important” were excluded

## Discussion

Our study aimed to determine, using the Delphi method of expert consensus, how to actively involve people with lived experience of suicide in suicide research. Of importance, our aim was to develop guidelines to support existing guidance regarding involvement of people with lived experience in health research more generally [4], including co-production [7]. These guidelines may be less applicable to other disciplines of suicide research which have a more robust tradition of qualitative methods studying lived experience, such as sociology, anthropology, or community psychology. These disciplines already have substantial methodological norms (e.g., “community-engaged research”) [40] and an established practice of leveraging and disclosing one’s own positionality in research and published material [41].

The lived experience and researcher panellists endorsed a total of 96 out of 126 statements (76%) across 17 domains, which were sourced from interview data and scientific and grey literature on consumer involvement in health research. As such, our results reflect a general agreement with recommendations on co-production and with the overarching principles and values of engagement and participation that support partnerships between researchers and people with lived experience of suicide [17].

The study panellists endorsed statements in the 17 proposed domains (sections), which span across the full research cycle: from deciding on the research question and securing funding, to conducting research and disseminating and implementing outcomes [4, 42]. From the co-production perspective, this implies development of lived experience leadership and capacity, involvement of people with lived experience in defining the problem, designing and delivering the solution, and evaluating the outcomes in suicide research [7]. In general, there was a strong consensus between the two study panels. In the Round One of rating, both panels agreed in their endorsement of 78 statements (68.4%) and rejection of seven statements (6.1%). This reflects agreement between people with lived experience of suicide and suicide researchers regarding specific “how to” recommendations regarding co-production and the broader co-production values reflected in the statements. Previous studies have also shown a high degree of agreement between views of people with lived experience of suicide and suicide researchers regarding research priorities [43, 44] and ethical issues in suicide prevention studies [45].

Two statements were endorsed unequivocally by the two panels. These were statements stipulating that lived experience researchers receive information and support on research ethics, privacy, and confidentiality, and a recommendation for academic researchers to present research data in understandable terms. The former statement is similar to a finding that attendance of appropriate research training is one of the key ethical issues in suicide prevention studies, a result of another Delphi expert consensus study involving suicide researchers and people with lived experience [45]. It can thus be recommended that people with lived experience who are actively involved in suicide research should be routinely provided with training on research ethics. The latter statement reflects a call for using “lay friendly language” [26; p. 623] when working with people with lived experience of suicide who are not familiar with research jargon.

Eight of 126 statements (6.3%) were rejected by both panels. These included statements in seven sections, including practical ways Research Institutions can support engagement of lived experience researchers, Representativeness and Diversity of Lived Experience, Collaboration and Co-production, Development of Collaborative Networks, Conduct of Research, Communication and Shared Decision, and Sharing of Power. This result may seem surprising as statements with a comparable content (e.g., engagement of an independent facilitator, people with lived experience piloting research measures and processes) are included in other guidance related to health research [13, 46]. It is possible that members of both panels already had experience of collaboration and found these recommendations unfeasible or burdensome. Further, unequivocal rejection of some of the proposed statements supports the original study rationale regarding the need for specific guidelines to support lived experience suicide research, as broader health-related research guidelines and frameworks are not automatically applicable to suicide research.

There were consistent discrepancies between the lived experience and researcher panels throughout the Delphi process, resulting in rejection of 15 statements (11.9%). The lived experience panel consistently endorsed and the researcher panel consistently rejected 14 statements across 11 domains. These included recommendations for researchers to contact their research organization or university to see if they have any funding for lived experience researcher(s) involvement, prior to the grant application being successful, involving more than one lived experience researcher in a research project, and ensuring that findings are available to public contributors, e.g., via open access journals. Differences between the two panels in our study may reflect an aspirational view of collaboration held by people with lived experience, which researchers more familiar with the reality of the academia find impractical or unfeasible given limited resources available, including budget for open access publications [47, 48]. Alternatively, these discrepancies may indicate the different needs and expectations of people with lived experience and suicide researchers, which may resurface and will need to be addressed in the process of research collaboration [49, 50].

Lived experience panellists endorsed two statements on training around language, which were rejected by research panellists. These statements recommended training in the language and terminology used in research for lived experience researchers and training in lay-friendly research language for academic researchers. It is possible that academic researchers do not experience language barriers in their communication with lived experience colleagues around research projects and/or believe that the established terminology used in their research domain is necessary to ensure precision and secure ongoing funding for research activities [51]. Another training-related statement rejected by academic researchers stipulated that they should seek insight into the lived experience of mental health issues outside the academic context. It is possible that expectations related to this statement exceed the boundaries of the established research and academic activities. Alternatively, the researcher panellists may believe that they have sufficient insight into the lived experience of suicide and additional exposure is not needed. Additionally, three of the statements rejected by research panellists related to aligning their research priorities and methods with lived experience researchers and organizations (sections on representativeness and diversity, managing expectations, and deciding on the research questions). Again, it is possible that there is a gap in the perception of this subject between academic and lived experience researchers: the former may believe that their activities meet the local and more general lived experience needs and expectations, while the latter still have unmet needs. Further research is needed to better understand the differences in ratings between the researcher and lived experience panellists in the field of suicide research and prevention [52].

On the other hand, the researcher panel consistently endorsed, and the lived experience panel consistently rejected, a statement related to support and self-care, which recommended that lived experience researchers are ready or in recovery to engage in the research process. This result may reflect the differences in the perceived importance and understanding of the concept of recovery between people with lived experience and researchers [53]. Further, the process of recovery in the context of fluctuating suicidal ideation and behavior is only an emerging research topic and its understanding is limited [54–57]. It also remains unclear how recovery can be defined in relation to suicide bereavement [58] and the caregiving role [59], highlighting the need for future work on more clarity around the meaning of these concepts.

### Strengths and limitations

Our study has a number of strengths. The study statements were derived from the literature and interview data. The study included two panels: a lived experience panel and a researcher panel, and the latter included researchers with lived experience of suicide. Panellists in both groups rated the proposed statements, and the voice of each panellist had equal weight in the endorsements. Further, both panels had an optimal number of panellists in all rounds (between 15 and 30 panellists) [31] with a very low attrition rate, especially in the researcher panel. This may indicate the importance of the topic and the panellists’ commitment to contribute to development of the guidelines and provide weight to the rationale and the findings of the study.

The findings must also be understood within the limitations of the study. The key concepts of the guidelines (“lived experience of suicide”, “co-production”) have been defined according to the literature and the current practice in suicide research and prevention in Australia and may be different from terminology used in other disciplines of suicide research or in other countries. The panellists self-identified as people with lived experience of suicide or suicide researchers, and we did not collect data on how long they were involved in research or the level of their experience in co-production. We did not collect data on details of the lived experience of suicide or on disciplines of suicide researchers. Given the networks used to recruit participants, it is most likely that the members of the research panel represented a wide range of professional backgrounds related to mental health and health, including psychology, psychiatry, mental health nursing, general practice, and social work. As such the guidelines may not reflect views of suicide researchers in other disciplines [60]. Further, it was beyond the scope of the study to review all methodological and epistemological approaches to co-production of knowledge. Future studies may also look at development of guidelines through the lens of knowledge production science.

## Conclusions

The consensus recommendations identified in our study provide a much-needed guide for researchers and people with lived experience of suicide on research collaboration and co-production. It is anticipated that the guidelines will contribute to a broader uptake of the co-production approach in suicide research in Australia and internationally. Future implementation and evaluation studies can determine the usefulness of these guidelines in progressing suicide prevention research. We are aware that it will take time to make co-production an integral part of the research process and we advise against an “all-or-nothing” approach, which may stall suicide research. Successful implementation and uptake of the guidelines will be facilitated by ongoing education of research institutions and funders about the value and the process of co-production, as well as providing training on co-production to researchers and people with lived experience of suicide.

## Electronic supplementary material

Below is the link to the electronic supplementary material.

Supplementary Material 1: Resources identified through grey literature searches

Supplementary Material 2: The endorsement ratings for each statement across the survey rounds

## Data Availability

All data generated or analysed during this study are included in this published article and its supplementary information files.
