# Supplementary material for: Active involvement of people with lived experience of suicide in suicide research: a Delphi consensus study
Source: BMC Psychiatry. 2023 Jul 11;23:496. doi: 10.1186/s12888-023-04973-9 (PMC10334649; doi:10.1186/s12888-023-04973-9)
Supplement: Supplementary file 1 — Supplementary Material 1: Resources identified through grey literature searches [file 12888_2023_4973_MOESM1_ESM.docx]

Appendix 1.

Resources identified through grey literature searches relevant to the development of Delphi survey statements on active involvement of people with lived experience of suicide in suicide research.

Ball S, Harshfield S, Carpenter A, Bertscher A, Marjanovic S. Patient and public involvement and engagement in research. Santa Monica: RAND Corporation. 2019.

Community Mental Health Drug and Alcohol Research Network (CMHDARN). Ask the Experts: A CMHDARN Best Practice Guide to Enabling Consumer and Carer Leadership in Research and Evaluation, Sydney, 2015.

Cooperative Research Centre for Living with Autism. Inclusive research practice guides and checklists for autism research: version 2. Brisbane, Queensland: Autism CRC Ltd. 2016.

Farr M, Davies R, Davies P, Bagnall D, Brangan E, Andrews H. A map of resources for co-producing research in health and social care. National Institute for Health Research (NIHR) ARC West and People in Health West of England; University of Bristol and University of West of England. Version 1.1, February 2020.

Loughhead M, Halpin M, Procter N. MHSPRG Lived Experience Engagement Framework [Internet]. UniSA; 2020 [cited 25 July 2021]. Available from: https://www.unisa.edu.au/contentassets/17bf3f604de44cf38e4aee6cf36e2a7f/mhsprg-le-engagement-framework-130220.pdf.

National Health and Medical Research Council and the Consumers Health Forum of Australia. The Statement on Consumer and Community Involvement in Health and Medical Research. Canberra: NHMRC. 2016.

National Institute for Health and Care Research [Internet]. Briefing notes for researchers – public involvement in NHS, health and social care research. NIHR; 2021 [cited 25 July 2021]. Available from: https://www.nihr.ac.uk/documents/briefing-notes-for-researchers-public-involvement-in-nhs-health-and-social-care-research/27371

National Institute for Health and Care Research [Internet]. Different experiences: A framework for considering who might be involved in research NIHR; 2021 [cited 25 July 2021]. Available from: https://www.nihr.ac.uk/documents/different-experiences-a-framework-for-considering-who-might-be-involved-in-research/27387

National Mental Health Consumer and Carer Forum. Advocacy brief: Co-design and co-production [Internet]. NMHCCF; 2017 [cited 25 July 2021]. Available from: https://nmhccf.org.au/our-work/advocacy-briefs/co-design-and-co-production.

National Mental Health Commission. Consumer and carer engagement: A practical guide. Sydney: NMHC. 2019.

Schrank B, Wallcraft J. Good practice guidance. In Wallcraft J, Schrank B & Amering M, editors. Handbook of service user involvement in mental health research. Sussex: Wiley-Blackwell; 2009. p. 243-47.

Strnadová I, Dowse L, Watfern C. Doing research inclusively: Guidelines for co-producing research with people with disability. Disability Innovation Institute UNSW Sydney. 2020.

Suicide Prevention Resource Center. Involving people with lived experience [Internet]. Oklahoma City, OK: SPRC; 2021 [cited 25 July 2021]. Available from: https://sprc.org/livedexperiencetoolkit/engagement

Suomi A, Freeman B, Banfield M. Framework for the engagement of people with a lived experience in program implementation and research. Black Dog Institute. 2017.
